# A critical juncture: Integrating large language models in biostatistical workflows

**DOI:** 10.1017/cts.2026.10713

**Published:** 2026-02-18

**Authors:** Vihaan Sahu

**Affiliations:** Medicine, Georgian National University SEU, Georgia

**To the Editor**

Grambow et al [1] provide essential, empirical insights into the integration of large language models (LLMs) in biostatistical workflows, revealing both rapid adoption and significant, unmitigated risks. Their survey of biostatisticians demonstrates substantial integration, with 63.8% (44/69) of respondents already using LLMs, nearly half (46.5%) daily [1]. The primary uses are for quantitative coding (77.5%) and writing tasks (76.3% for editing), indicating a profound shift in daily practice [1]. However, this adoption starkly outpaces the development of adequate safeguards. The most critical finding is that 70.7% (29/41) of users reported encountering incorrect LLM outputs with potentially serious consequences, spanning incorrect code generation, statistical misinterpretation, content fabrication, and inappropriate tone [1].

This scenario represents a genuine paradigm shift. LLMs are not merely augmenting workflows but are fundamentally transforming how biostatisticians generate code, communicate findings, and access knowledge, creating a new, hybrid human-AI workflow [2,3]. This transformation necessitates a parallel shift in our approach to verification and quality control. The current reliance on individualized, ad hoc verification strategies such as personal expertise, external checks, and debugging is insufficient [1]. This is compounded by a glaring institutional support gap: while 49.3% of respondents reported organizational encouragement to use LLMs, only 18.8% had access to formal guidance or training [1].

This disconnect between rapid adoption and inadequate safeguards opens a larger series of essential experiments. First, we need rigorous, longitudinal studies to examine how LLM integration affects the reproducibility and quality of clinical research analyses, as initial comparative assessments show alarming variability in endpoint calculations like objective response rate [4]. Second, domain-specific verification frameworks must be developed and validated to systematically detect the high-frequency error types identified [1,5]. Third, comparative evaluations of different LLM architectures and prompting strategies for core biostatistical tasks are urgently required, moving beyond simple accuracy metrics to assess reasoning, hallucination rates, and robustness as recommended by scoping reviews [6,7].

The impact on clinical and translational science is twofold. Properly managed, LLMs could accelerate trial design, analysis, and the communication of complex results. However, the current high error rate and lack of standardized verification pose a direct threat to research integrity and subsequent clinical decision-making [1,8,9]. The strong demand for structured support (75.9% of respondents wanted case studies and 69.0% interactive tutorials) must be met with field-specific resources that emphasize critical evaluation and the eight core principles for responsible use proposed by the authors, including collaborative verification and transparency [1,10].

Grambow et al [1] have laid a crucial evidentiary foundation. Their work should catalyze a coordinated effort to develop the specialized training, robust evaluation frameworks, and institutional policies required to ensure this paradigm shift enhances, rather than undermines, the methodological rigor that is the cornerstone of valid clinical and translational science.
